# Pelvic Organ Prolapse Repair Using Robotic-Assisted Sacral Hysterocolpopexy vs Vaginal Surgery with the Uphold™ System: 1-Year Clinical Outcomes

**DOI:** 10.1007/s00192-024-06017-6

**Published:** 2025-01-08

**Authors:** Georgios Poutakidis, Christian Falconer, Daniel Altman, Ulrika Johannesson, Anju Zhang, Charlotta Ericson, Mats Stenberg, Sabine Altrock, Edward Morcos

**Affiliations:** 1 Department of Clinical Sciences, Division of Obstetrics and Gynecology, Karolinska Institutet Danderyd Hospital, SE- 182 88 Stockholm, Sweden; 2 Department of Obstetrics and Gynecology, Danderyd University Hospital, SE- 182 88 Stockholm, Sweden; 3 Department of Women’s and Children’s Health, Uppsala University, Uppsala, Sweden; 4 Stockholm Urogynecological Clinic, Stockholm, Sweden; 5 Department of Clinical Science and Education, Södersjukhuset, Karolinska Institutet and the Division of Obstetrics and Gynecology at Södersjukhuset, Stockholm, Sweden; 6 Department of Obstetrics and Gynecology, Västerås Central Hospital, Västerås, Sweden

**Keywords:** Apical prolapse surgery, Robotic-assisted sacral hysterocolpopexy, Uphold™ vaginal mesh, Safety, Anatomical and patient-reported prolapse outcomes, Effectiveness

## Abstract

**Introduction and Hypothesis:**

The aim of the study was to compare clinical outcomes when using robotic-assisted sacral hysterocolpopexy (RASC) and vaginal surgery using the Uphold™ Vaginal Support System mesh for pelvic organ prolapse repair.

**Methods:**

This was a nonrandomized, prospective, multicenter study in which 72 women underwent RASC, and 73 Uphold™ surgery, for apical prolapse (POP-Q C ≥ stage II). Anatomical outcomes were assessed using the Pelvic Organ Prolapse Quantification (POP–Q) system. Subjective outcomes were evaluated using the Pelvic Floor Distress Inventory 20 (PFDI-20), the Pelvic Floor Impact Questionnaire – short form (PFIQ-7), and the Pelvic Organ Prolapse/Urinary Incontinence Sexual Questionnaire (PISQ-12), as well as pain estimation using the visual analog scale (0–10).

**Results:**

One year after surgery, an optimal apical segment outcome (POP-Q C stage 0–1) was achieved in 96.4% and 93.3% for the RASC and Uphold™ respectively, *p* = 0.49. However, reoperation for prolapse recurrence was significantly more common after RASC (11 out of 72 [15.3%] vs Uphold™ (2 out of 71 [2.8%], *p* = 0.005), and an optimal outcome of the anterior vaginal wall was higher after Uphold™ (*p* < 0.001). Postoperative PFDI-20, PFIQ-7, and pain significantly improved for both RASC and Uphold™ (*p* = 0.004 to < 0.001), but a more pronounced improvement in the total PFDI-20 and POPDI-6 sub-scores was observed after Uphold™ than after RASC (−73 ± 55.6 vs −49.2 ± 43.7, *p* = 0.005 and −39.6 ± 23.6 vs −27 ± 23.9, *p* < 0.001 respectively).

**Conclusions:**

Reoperation for prolapse recurrence within 1 year was more common after RASC than after Uphold™. However, the rate of complications was low overall and there were few and largely insignificant differences in outcomes when comparing RASC and Uphold™.

## Introduction

Sacral colpopexy involves the use of polypropylene mesh to support the uterus, or the vaginal apex, by attaching it to the sacral promontory [1]. Sacrocolpopexy has been considered a standard procedure for apical pelvic organ prolapse repair and is associated with success rates within the range 58% to 100%, with a reoperation rate of 0% to 18.4% [2–4]. A minimally invasive approach using laparoscopy for sacral hysteropexy/colpopexy (LSHP/LSC) and the robotic-assisted sacral colpopexy (RASC) has lowered the morbidity associated with the procedure compared with the open abdominal approach [5, 6]. The use of robotic-assisted surgery has grown since the advent of better wrist dexterity, a 3D view, and motion scaling [6, 7]. However, the efficacy of RASC and LSHP/LSC is comparable as indicated in previous studies, but the only difference is less bleeding with the RASC [6–8].

Before removal from the market, the Uphold™ LITE Vaginal Support System was associated with significantly improved subjective and anatomical outcomes at long-term follow-up [9–11]. The product used a small amount of polypropylene, compared with other transvaginal mesh products, and previous studies suggest that the use of the Uphold™ LITE Vaginal Support System might be efficacious with relatively low rates of complications when performed at centers with a high surgical volume [10–16]. Although the Uphold™ LITE Vaginal Support System is no longer commercially available, continued research on the use of polypropylene mesh implants has been encouraged by health authorities in order to collect long-term clinical safety and efficacy data [9].

The aim of the present study was to compare the short-term safety, clinical outcomes, and effectiveness following the use of robotic-assisted sacral hysterocolpopexy (RASC) and the Uphold™ LITE Vaginal Support System.

## Materials and Methods

### Patient and Surgery

This was a parallel cohort study aimed at including 100 patients undergoing both surgical procedures between 1 January 2018 and 31 December 2019, at three participating clinics in Stockholm and Västerås, Sweden. All patients had symptomatic apical prolapse (uterine or vaginal vault, Pelvic Organ Prolapse Quantification [POP-Q] C stage ≥ 2) with or without other symptomatic concomitant vaginal compartment prolapse. Allocation to surgical procedure was dependent on the surgeon, but only postmenopausal women were included in the Uphold™ cohort. Exclusion criteria included current or previously treated pelvic organ cancer; cervical elongation; severe rheumatic disease; and connective tissue disorder. Clinical follow-up visits were performed 3 months and 1 year after surgery.

The RASC procedure was performed by three experienced robotics surgeons, who had performed at least 30 RASC procedures, using the Da Vinci surgical system (Intuitive Surgical, Sunnyvale, CA, USA) and an established technique [17–20]. The Artisyn® Y-Shaped Mesh (Ethicon) or the Restorelle® XL Mesh (Coloplast) were adjusted to each patient individually by the operating surgeon [17–20]. After exposure of the peritoneum of the sacral promontory, the pelvic sidewall was dissected, as well as the anterior vaginal wall and the bladder to the trigone area to be able to fix the anterior part of the mesh to the anterior vaginal wall. The posterior mesh was fixed to the posterior vaginal wall after dissection. The apical vaginal part of the mesh was fixed to the posterior wall of the cervix if the uterus was preserved (hysteropexy), to the cervix stump if a previous subtotal hysterectomy (supra-cervical hysterectomy) was done or if a subtotal hysterectomy was done at the same time at which the mesh was operated on, or to the vaginal vault (vaginal sacrocolpopexy) if women had had a previous total hysterectomy. The mesh was then passed just above the upper part of the cardinal ligament, around the cervix on each side, to be further fixed down to the anterior vaginal wall at a maximum length of 4 cm depending on the size of the anterior vaginal wall defect. The mesh was placed down on the anterior longitudinal ligament at the sacral promontory, medially to the right ureter, avoiding the hypogastric nerve [17–20]. Nonresorbable stitches was used for the mesh fixation. The peritoneum was closed with resorbable running sutures, finally, to cover the mesh.

Using a standardized surgical procedure, the Uphold™ LITE Vaginal Support System was used in all patients, as previously described [10, 11, 15, 16, 21]. The Uphold™ LITE mesh is a monofilament, microporous, and uncoated polypropylene mesh. Through a midline incision in the anterior vaginal wall the Uphold™ LITE vaginal mesh was inserted in order to suspend the apical vaginal compartment (uterus or vaginal vault) to the sacrospinous ligament level at both sides using the Capio™ SLIM Suture Capturing Device (Boston Scientific) [15, 16, 21]. Additional prolapse surgery using native-tissue repair included anterior and posterior colporrhaphies with, or without, perineorrhaphy.

All patients in the two cohorts received prophylactic intravenous antibiotics. No midurethral sling (MUS) procedures for stress urinary incontinence were performed at the time of primary surgery in the RASC or the Uphold™ cohorts.

### Anatomical Quantification by the POP-Q

The POP-Q system was used for anatomical assessment of prolapse; POP-Q C for the apical vaginal segment, POP-Q Ba for the anterior vaginal wall, POP-Q Bp for the posterior vaginal wall, and POP-Q TVL for assessment of the total vaginal length [22]. All women had symptomatic apical prolapse (POP-Q stage ≥ 2). POP-Q stage 0 or 1 in the apical vaginal segment was considered an optimal anatomical outcome after surgery and was defined as the primary outcome measure.

### Subjective Pelvic Floor Outcomes

To assess subjective outcomes, the Pelvic Floor Distress Inventory 20 (PFDI-20) questionnaire and three subscales: Pelvic Organ Prolapse Distress Inventory-6 (POPDI-6), Urinary Distress Inventory-6 (UDI-6), and Colorectal-Anal Distress Inventory–8 (CRADI-8) were used [23]. The total score of each subscale ranges from 0 to 100 points; the total PFDI-20 is the sum of the three scales and ranges from 0 to 300 points. Each individual question of the POPDI-6 and UDI-6 ranges from 0 to 16.6 points out of a maximal score of 100 points per scale. The PFDI-20 Question 3 was used to assess the degree of sensation or visualization of a vaginal bulge and a score of 0–1 point indicates subjective success, i.e., no sense of vaginal bulge after prolapse repair. The PFIQ-7 includes the Urinary Impact Questionnaire (UIQ-7), the Colorectal-Anal Impact Questionnaire (CRAIQ-7), and the Pelvic Organ Prolapse Impact Questionnaire (POPIQ-7) [23]. To measure the impact on patients’ sexual function, the Pelvic Organ Prolapse/Urinary Incontinence Sexual Questionnaire (PISQ-12) was used, which includes 12 questions concerning behavioral/emotional, physical, and partner-related sexual domains [24]. The visual analog scale (VAS) was used to assess the degree of pain [25]. The VAS scale is a horizontal 11-point scale, where 0 indicates no pain and 10 points indicate severe pain.

### Statistical Analyses

A two-sided independent sample *t* test was used to compare independent variables. For analysis of the statistical difference between nominal data we used the Chi-squared test of independence. Fisher’s exact test was used for analysis of the categorical data when classifying results in two different ways in order to examine the significance of the association, i.e., the contingency between the two kinds of classification. McNemar's test was used for analysis of the paired nominal data. Kendall's *W* test (Kendall's coefficient of concordance) was used to test the nonparametric statistic for rank correlation in order to assess the agreement among raters and in particular the inter-rater reliability over the time of the follow-ups. Repeated measures ANOVA was used to test total values and to compare continued variables and differences in changes over time of follow-up between the two surgery cohorts. The Bonferroni post hoc test was used to limit the apparent statistical significance when analyzing the effect of multiple comparison follow-ups in the dependent variable. The Independent-Samples Proportions test was used to statistically test and provide confidence intervals for the difference in two independent binomial proportions. Linear regression analysis was used to test the correlation between the dependent and independent variables. Multiple regression analysis was used to test the linear correlation between multiple independent variables (demographic characteristics) and dependent variables (POP-Q, PFDI-20, and POPDI-6). Also, we used the logistic regression analysis as a predictive analysis of the coefficient of linear combination in categorical dependent variables. A post hoc power analysis concluded that a minimum of 67 individuals in each cohort (total 67*2 = 134) yielded 80% power to detect an association of the outcomes in POP-Q, PFDI-20, PFIQ-7, PISQ-12, and VAS scores between the cohorts when we use a linear regression model (two predictors) with an effect of Cohen’s f = 0.39 (medium effect). All missing data were considered missing without imputation of data, i.e., we used per protocol statistical analysis. Statistical analysis was performed using predictive analysis software (IBM@SPSS© Statistics, Version 28, 2017; SPSS, Chicago, IL, USA).

## Results

### Demographics, Medical, and Surgical Characteristics

The study did not reach the predetermined aim of including 100 patients in each arm, yet it achieved the post hoc power assumption. Following the FDA warning on the use of vaginal mesh in May 2019, the study was closed and only 72 patients in the RASC cohort and 73 patients were included in the Uphold™ cohort.

Preoperative demographics, medical, and surgical characteristics are described in detail in Table 1. The mean age was 10 years younger in the RASC cohort than in the Uphold™ cohort (58.8 ± 13.1 [33–77 years] vs 69.5 ± 7.9 [51–83 years] respectively; *p* < 0.001). Postmenopausal status was reported by 64.5% of women in the RASC cohort compared with 98.6% in the Uphold™ cohort (*p* < 0.001). Having had previous pelvic floor surgery was significantly less common in the RASC than in the Uphold™ cohort (*p* = 0.005) but there were no significant differences between the cohorts with regard to previous hysterectomy.

**Table 1:** Demographics, medical, and surgery characteristics

	RASC			Uphold™			
	Mean ± SD, *n* (%)	CI	*n*	Mean ± SD, *n* (%)	CI	*n*	*p* value
Age (years)	58.8 ± 13.1 (33–77)	55.7–61.9	72	69.5 ± 7.9 (51–83)	67.7–71.4	73	< 0.001
Length (cm)	167.7 ± 6.3	166.2–169.2	72	164.7 ± 6.2	163.3–166.2	73	0.009
Weight (kg)	70.5 ± 11.1	67.8–73.1	72	69.4 ± 9.2	67.2–71.5	73	0.546
BMI (kg/m^2^)	25.0 ± 3.7	24.2–25.9	72	25.6 ± 3.1	24.8–26.3	73	0.396
Parity	2.4 ± 1.0	2.2–2.7	61	2.9 ± 10.9	2.43–2.9	72	0.190
Vaginal deliveries	2.3 ± 1.0	2.0–2.5	61	2.65 ± 0.9	2.45–2.9	70	0.027
Caesarean section	0.1 ± 0.5	0.02–0.3	61	0.07 ± 0.4	–0.02–1.7	70	0.330
Menopause	40 (64.5%)	(52.2–75.5%)	62	72 (98.6%)	(93.8–99%)	73	0.001
No hormonal therapy	33 (54.1%)	(41.6–66.2%)	61	33 (45.2%)	(34.2–56.6%)	73	0.083
Estrogen (vaginal)	20 (32.3%)	(22.0–45.1%)	61	37 (50.7%)	(39.4–61.9%)	73	
Estrogen (tablet)	1 (1.6%)	(0.2–7.4%)	61	2 (2.7%)	(0.6–8.5%)	73	
Estrogen (vaginal and tablet)	2 (3.3%)	(0.7–10.1%)	61	1 (1.4%)	(0.1–6.2%)	73	
Hormonal IUD	5 (8.2%)	(3.2–17.0%)	61	0	0	73	
Smokers	4 (6.5%)	(2.2–14.6%)	62	6 (8.2%)	(3.5–16.2%)	73	0.752
Physical training							
0 times/week	12 (19.7%)	(11.2–30.9%)	61	22 (30.1%)	(20.5–41.3%)	73	0.239
Once/week	24 (39.3%)	(27.8–51.9%)		30 (41.1%)	(30.3–52.5%)		
2–4 times/week	25 (41%)	(29.3–53.5%)		21 (28.8%)	(19.4–39.8%)		
Somatic diseases							
None	25 (34.7%)	(24.5–46.1%)	72	10 (13.7%)	(7.3–22.9%)	66	0.001
CVS	7 (9.9%)	(4.3–17.7%)	71	21 (31.8%)	(19.4–39.8%)	66	0.001
Thyroid dysfunction	16 (22.2%)	(13.8–32.8%)	72	9 (12.3%)	(6.3–21.3%)	73	0.091
Asthma	18 (25.0%)	(16.1–35.8%)	72	13 (17.8%)	(10.4–27.7%)	73	0.248
Arthrosis	18 (25.0%)	(16.1–35.8%)	72	13 (17.8%)	(10.4–27.7%)	73	0.180
Diabetes	3 (4.2%)	(1.2–10.7%)	72	4 (5.5%)	(1.9–12.5%)	73	0.719
Rheumatism	6 (10.0%)	(4.3–19.5%)	60	8 (11.8%)	(5.7–21%)	68	0.959
Other	27 (37.5%)	(27.0–49.0%)	72	19 (26%)	(17–36.9%)	73	0.243
Previous pelvic floor surgery							
None	30 (52.4%)	(40.2–64.4%)	62	20 (28.6%)	(19–39.9%)	70	0.005
Prolapse	19 (30.6%)	(20.2–42.8%)	62	38 (53.3%)	(42–64.8%)	71	0.006
Hysterectomy	21 (29.2%)	(19.6–40.3%)	72	15 (20.5%)	(12.5–30.8%)	73	0.270
Hysterectomy and prolapse	6 (8.3%%)	(3.6–16.4%)	72	5 (6.8%)	(2.7–14.4%)	73	0.772
Urinary incontinence	5 (8.1%)	(3.1–16.8%)	62	7 (10%)	(4.6–18.6%)	70	0.678
Hysterectomy and prolapse and urinary incontinence	1 (1.6%)	(2.1–17.9%)	62	1 (1.4%)	(0.1–6.2%)	73	0.999
Pain (VAS 0–10)	2.8 ± 2.75	2.1–3.4	67	2.2 ± 2.3	1.6–2.7	68	0.162
Operative bleeding (ml)	24.9 ± 52.6	12.5–37.2	70	64.2 ± 72.6	47.2–81.1	73	< 0.001
Operating time (min)	150.8 ± 36.1	142.4–159.3	72	68 ± 26	62–74	73	< 0.001
Hospital stay (days)	1.1 ± 0.5	1–1.3	71	1.3 ± 1.32	0.95–1.57	73	0.921

Independent sample two–sided *t* test, Chi–squared test of independence, and Fisher´s exact test were used. *p* < 0.05 was considered significant

### Surgery, Complications, and Re-interventions

Seventy-two patients were operated on in the RASC cohort including hysteropexy, i.e., preserving the uterus (*n* = 44), subtotal hysterectomies with fixation of the cervix stump (*n* = 7), and vault sacrocolpopexy, i.e., vaginal vault prolapse after previous total hysterectomy (*n* = 21). Concurrent surgical procedures included supracervical hysterectomy (*n* = 7), bilateral salpingo-oophorectomy (*n* = 2), and perineorrhaphy (*n* = 7).

Seventy-three patients underwent the Uphold™ procedure for uterine (*n* = 58) and vaginal vault prolapse (*n* = 15). Additional native tissue repair procedures for symptomatic prolapse were performed in 36 patients, including perineorrhaphy (*n* = 36) and posterior colporrhaphy (*n* = 26).

All patients in the RASC cohort received general anesthesia, whereas 66 out of 73 patients (90.4%) received spinal anesthesia and 7 out of 73 (9.6%) received general anesthesia in the Uphold™ cohort. The operating time was longer in the RASC cohort than in the Uphold™ cohort (151 ± 36.1 vs 68 ± 26 minutes, *p* < 0.001), but there was no significant difference with regard to duration of hospital stay between the cohorts.

All complications are presented in Table 2. The low rate of complications did not allow for statistical analysis of surgical complications according to the Clavien–Dindo system. Total number of reoperations for prolapse, incontinence, or mesh revision during the 1-year follow-up was higher after RASC than after Uphold™ (15 out of 72 patients (20.8%) and 5 out of 73 patients [6.8%], *p* = 0.018). Symptomatic prolapse recurrence needed secondary reoperation in the RASC cohort, including 5 apical, 7 anterior vaginal wall, and 8 posterior vaginal wall prolapses. Thus, 12 patients were re-operated for prolapse recurrence in the RASC cohort (4 sacrospinous ligament fixation, 1 uterosacral ligament suspension, 7 perineorrhaphy, 7 anterior colporrhaphy, 8 posterior colporrhaphy), 3 patients underwent mesh revision and 2 patients had received a MUS for stress urinary incontinence. Other surgical re-interventions in the RASC cohort included 2 diagnostic laparoscopies and 1 for umbilical hernia. Symptomatic prolapse recurrence needed secondary reoperation in the Uphold™ cohort, including 2 posterior wall prolapses and 1 cervical descensus. Thus, a total of 5 patients in the Uphold™ cohort underwent secondary procedures, including 2 cases of posterior colporrhaphy, 1 case of cervical resection, and 2 MUS for stress urinary incontinence. In 1 patient the Uphold™ mesh was extracted 6 days after the primary surgery because of postoperative pelvic pain. Mesh exposure was reported for 3 patients at the RASC cohort, ranging between 1 and 1.5 cm and mesh revision was carried out at the operation theater whereas mesh exposure was reported in 2 cases in the Uphold™ cohort ranging between 3 and 5 mm and were managed by a simple cut at the outpatient clinic.

**Table 2:** Complication and reoperation: intraoperative and the primary care occasion, postoperative period (hospital departure to 3 months), and total, i.e., from primary surgery to 1 year after surgery

	RASC (*n* = 72)	Uphold™ (*n* = 73)			
	*n* (%)	*n* (%)	Risk ratio	CI	*p* value
Intraoperative and primary care occasion					
Bladder, urethra or rectum perforation	None	None	–	–	–
Ureter injury	1 (1.4%)	None	–	–	–
Hematoma	1 (1.4%)	1 (1.4%)	1.01	0.1–15.6	0.992
Bleeding ≥ 500 ml	None	None	–	–	–
Bladder–emptying difficulties^a^	None	3 (4.1%)	–	–	–
Re–operation (primary operative care occasion)	2 (2.8%)^a^	1 (1.4%)^d^	2.03	0.1–14.5	0.568
Urinary tract infection	None	2 (2.7%)	–	–	–
Postoperative up to 3 months					
Re-admission	3 (4.2%)	2 (2.7%)	0.99	0.1–6.9	0.854
Wound infection	1 (1.4%)	1 (1.4%)	1.01	0.1–15.8	0.992
Reoperation	2^c^ (2.8%)	None	–	–	–
Urinary tract infection	4 (5.6%)	13 (18.1%)	0.21	0.2–1.7	0.002
Antibiotics for UTI	4 (5.6%)	12 (16.4%)	0.34	0.2–1.8	0.034
Total (1 year)					
Prolapse reoperation	12 (16.7%)	2 (2.8%)	4	0.5–6.2	0.015
Urinary incontinence surgery (MUS)	2 (2.8%)	2 (2.94%)	0.94	0.1–6.7	0.932
Pelvic floor insufficiency reoperation^b^	15 (20.8%)	5 (6.9%)	3	0.6–4.2	0.018
Any reoperation	17 (23.6)	5 (6.9%)	3.4	0.7–4.4	0.004
Any complication	25 (34.7%)	25 (34.3%)	0.94	0.3–2.2	0.932
Urinary tract infection	5 (6.9%)	21 (29.2%)	0.24	0.3–1.4	0.002

Independent-samples proportion test was used to test the statistical difference between the cohorts and provide confidence intervals (CI) if available. *p* < 0.05 was considered significant

^a^Diagnostic laparoscopy, hematoma

^b^Reoperation for prolapse, urinary incontinence, and mesh revision

^c^Reoperation for umbilical hernia, diagnostic laparoscopy for suspected ileus

^d^Mesh extraction

### Anatomical Outcomes

Anatomical outcomes evaluated by the POP-Q system are described in Table 3. At the preoperative examination, all patients had an apical segment prolapse, i.e., POP-Q C ≥ −1 (POP-Q C, stage 2–4), and there was no significant difference between the cohorts (*p* = 0.54). Cystocele (POP-Q Ba ≥ 0) was overrepresented in the Uphold™ cohort compared with the RASC cohort (*p* < 0.001), whereas rectocele (POP-Q Bp ≥ 0) was overrepresented in the RASC cohort compared with the Uphold™ cohort (*p* = 0.014).

**Table 3:** Anatomical outcomes estimated by Pelvic Organ Prolapse Quantification (POP–Q; preoperative, 3 months, and 1 year after surgery)

	Preoperative			3 months			1 year			Success rate *n* (%)		Success rate *p* value	
	RASC	Uphold™		RASC	Uphold™		RASC	Uphold™		RASC	Uphold™	^a^Between Cohort	^b^Between changes
	Mean ± SD	Mean ± SD	*p* value	Mean ± SD	Mean ± SD	*p* value	Mean ± SD	Mean ± SD	*p* value	*n* (%)	*n* (%)	RASC vs Uphold™	RASC vs Uphold™
*n*	72	73		53	66		55	62		55	62	55 vs 62	55 vs 62
Apical													
C	1.6 ± 2.5	1.9 ± 2.7	0.413	−5.5 ± 2.8	−5.4 ± 1.7	0.762	−5.6 ± 2	−5.4 ± 2.4	0.531	53/55 (96.4)	58/62 (93.5%)	0.683	0.491
Anterior wall													
Aa	1.5 ± 1.9	2.4 ± 1	< 0.001	−1.6 ± 1.9	−2.8 ± 0.6	< 0.001	−1.9 ± 1.6	−2.5 ± 1.3	0.028	36/55 (67.9%)	57/62 (91.9%)	< 0.001	< 0.001
Ba	2 ± 2.3	3.7 ± 2.1	< 0.001	−1.5 ± 2.3	−2.8 ± 0.6	< 0.001	−2.1 ± 1.4	−2.4 ± 1.6	0.039	40/53 (75.5%)	57/62 (91.9%)	0.015	< 0.001
D	−2.9 ± 4.1	−3.5 ± 3.8	0.457	−5.2 ± 5.3	−6.7 ± 1.6	0.138	−6.6 ± 1.5	−6.2 ± 2	0.229				
Posterior wall													
Ap	−0.5 ± 2.1	−0.9 ± 1.6	0.173	−2.5 ± 1.3	2.6 ± 0.8	0.544	−2.1 ± 1.3	−2.4 ± 1.3	0.322	39/52 (75%)	51/62 (83.9%)	0.344	0.404
Bp	−0.2 ± 2.7	−1.1 ± 2.5	0.044	−2.3 ± 1.5	−2.6 ± 1	0.050	−2.3 ± 1	−2.4 ± 1.1	0.462	41/52 (78.8%)	52/62 (83.9%)	0.491	0.290
Gh	5.1 ± 1.1	5 ± 0.9	0.659	4.8 ± 1	4.1 ± 0.6	< 0.001	4.7 ± 0.8	4.3 ± 0.6	0.001				
	2.3 ± 0.8	2.2 ± 0.8	0.883	2.5 ± 0.7	2.97 ± 0.6	< 0.001	2.6 ± 0.5	2.8 ± 0.5	0.146				
Pb TVL	9.4 ± 1.0	9.3 ± 1	0.312	9.1 ± 1.3	9 ± 1	0.621	9.2 ± 1	8.7 ± 1.2	0.032				
Cystocele (Ba ≥ 0)	59 (81.9%)	72 (98.6%)	< 0. 001	14 (29.8%)	2 (3%)	< 0. 001	9 (16.7%)	5 (8.1%)	0. 156				
Rectocele (Bp ≥ 0)	33 (47.1%)	20 (27.4%)	0.015	6 (13.3%)	3 (4.5%)	0.157	5 (9.6%)	6 (9.7%)	0.991				

McNemar's test was used for the analysis.* p* value < 0.05 was considered significant

^a^Between cohort = between cohort score

^b^Between changes in the cohort = between changes, i.e., difference in the cohort scores from the preoperative to the follow-up

At the 1-year follow-up, 55 patients (76.4%) in the RASC cohort and 62 patients (85%) in the Uphold™ cohort were available for a physical examination. The POP-Q score was significantly improved for all vaginal compartments at the 1-year follow-up within the group difference, i.e., in each cohort separately. The apical POP-Q C improved from 1.6 ± 2.5 to −5.6 ± 2 for the RASC cohort and from 1.9 ± 2.7 to −5.4 ± 2.4 for the Uphold™ cohort (*p* < 0.001). This corresponded to a significant number of patients defined as anatomical success, i.e., POP-Q stage 0–1 at the 1-year follow-up for both surgical cohorts when compared with baseline (*p* = 0.002 for RASC and *p* < 0.001 for Uphold™). Comparative analysis of the success rates of the apical and posterior wall POP-Q points and changes over time (preoperatively to 1 year) showed no difference between the RASC and Uphold™ groups (Table 3). However, there was a significantly higher success rate for the anterior wall POP-Q score for the Uphold™ compared with the RASC cohort (*p* = 0.015, *p* < 0.001).

Excluding re-operated patients for prolapse recurrence (10 patients in the RASC group and 2 patients at the Uphold™ group), we did the same analysis of the 1-year POP-Q outcome after the RASC and the Uphold™ (*n* = 45 and *n* = 60 respectively). Results when excluding prolapse re-operated patients were identical to results when the 1-year POP-Q total patients presented for the physical examination (RASC *n* = 55 and Uphold™* n* = 62). In detail, there was only a significant difference for improvement in the anterior vaginal wall outcome (POP-Q, Ba stage 0–1) at the Uphold™ cohort (55 out of 60 [91.7%]) compared with the RASC cohort (*n* = 34 out of 45 [75.6%], *p* = 0.022). However, no significant difference was found in the apical segment outcome (POP-Q, C stage 0–1) between the cohorts (43 out of 45 [95.6%] for the RASC group and 56 out of 60 [93.3%] for the Uphold™ group, *p* = 0.347) or the posterior vaginal wall POP-Q outcome (POP-Q, Bp stage 0–1; 34 out of 44 [77.3%] for the RASC group and 50 out of 60 [93.3%] for the Uphold™ group, *p* = 0.425).

### Subjective Pelvic Floor Outcomes

Subjective outcomes are presented in Tables 4 and 5. Improvement in subjective pelvic floor outcomes i.e., reduced scores of the total PFDI-20 and PFIQ-7 and subscales were significant over follow-up for both two cohorts (Table 4). There was no significant difference between the RASC cohort and the Uphold™ cohort in the total PFDI-20 and PFIQ-7, as well as subscales except for the POPDI-6 at 3 months and 1 year after surgery which was significantly lower in the Uphold™ cohort (*p* = 0.015 and *p* = 0.003 respectively) (Table 4).

**Table 4:** Patient–reported pelvic floor outcomes (Pelvic Floor Distress Inventory 20 [PFDI–20], Pelvic Floor Impact Questionnaire – short form [PFIQ–7], and Pelvic Organ Prolapse/Urinary Incontinence Sexual Questionnaire [PISQ–12]), preoperatively, 3 months, and 1 year after surgery

	Preoperatively					3 months					1 year					ANOVA repeated measures	
	RASC *mean* ± *SD*	*n*	Uphold™ *mean* ± *SD*	*n*	*P*–value	RASC *mean* ± *SD*	*n*	Uphold™ *mean* ± *SD*	*n*	*P*–value	RASC *mean* ± *SD*	*n*	Uphold™ *mean* ± *SD n*	*n*	*P*–value	RASC *P*–value	Uphold™ *P*–value
PFDI-20 total	114.1 ± 52.14	64	125.5 ± 54.8	74	0.015	61.5 ± 45.1	59	56.3 ± 39.7	65	0.651	60.3 ± 42.6	63	49.6 ± 35.1	64	0.128	< 0.001	< 0.001
POPDI–6	49.0 ± 21.70		51.6 ± 21.6		0.486	19.6 ± 16.9		13 ± 12.9		0.017	19.6 ± 17.1		10.9 ± 12.8		0.002	< 0.001	< 0.001
CRADI–8	25.0 ± 17.87		28.6 ± 20.1		0.262	18.4 ± 17.6		18.3 ± 18.2		0.699	18.7 ± 17.7		17.3 ± 16		0.218	0.002	< 0.001
UDI–6	40.2 ± 25.40		45.3 ± 26.4		0.255	23.5 ± 21.8		24.9 ± 20.9		0.002	22.0 ± 21.0		21.4 ± 17.6		0.492	< 0.001	< 0.001
PFIQ–7 total	54.9 ± 55.6	62	61.9 ± 55.7	72	0.469	17.7 ± 24.9	58	18.9 ± 25.2	66	0.796	20.3 ± 29.8	61	21.2 ± 41.1	64	0.896	< 0.001	< 0.001
PFIQ–UIQ-7	19.1 ± 23.6		25.9 ± 24.7		0.107	10.9 ± 17		11.5 ± 17.2		0.836	8.7 ± 14.2		9.6 ± 17.1		0.754	0.032	< 0.001
PFIQ–CRAIQ-7	22.6 ± 23.9		14.1 ± 20.8		0.795	6.8 ± 13.9		11.6 ± 18.1		0.490	5.6 ± 11.8		7.8 ± 17.6		0.572	0.014	0.002
PFIQ–POPIQ-7	13.2 ± 18.7		21.9 ± 22.5		0.860	9.7 ± 15.5		7.3 ± 13.4		0.828	6.3 ± 12.1		3.9 ± 12.6		0.439	< 0.001	< 0.001
PISQ–12 total	25.4 ± 11.3	49	26.1 ± 12.4	52	0.785	26.5 ± 14.7	44	20.9 ± 15.9	39	0.098	23.8 ± 14.8	39	27.1 ± 13.9	40	0.306	0.332	0.004
PISQ-12–behavioral	7.3 ± 4.3	49	7.2 ± 4.6	52	0.898	8.2 ± 4.7	43	6.1 ± 4.9	38	0.053	6.2 ± 4.6	39	6.8 ± 4.6	40	0.564	0.003	0.773
PISQ-12–physical	13.0 ± 4.7	43	14.3 ± 4.9	47	0.189	14.9 ± 4.3	36	14 ± 5.7	28	0.485	15.2 ± 4.7	35	16 ± 5.8	32	0.528	0.005	0.132
PISQ–12-partner	7.5 ± 2.2	44	6.9 ± 2.6	45	0.221	8.1 ± 2.3	34	6.8 ± 3.3	28	0.059	7.5 ± 3.1	33	7.2 ± 3	29	0.720	0.207	0.089

Independent sample two–sided *n* test was used to compare pelvic floor outcomes (PFDI–20, PFIQ–7, PISQ–12, and subscales) between the two cohorts of surgery at the time of measurement (preoperatively or 3 months or 1 year after surgery). ANOVA repeated measures was used for statistical analysis over time (preoperatively, 3 months, and 1 year after surgery) for the two surgery cohorts separately

*POPDI-6* Pelvic Organ Prolapse/Urinary Incontinence Sexual Questionnaire-6, *CRADI-8* Colorectal-Anal Distress Inventory-8, *UDI-6* Urinary Distress Inventory-6, *UIQ-7* Urinary Impact Questionnaire-7, *CRAIQ-7* Colorectal-Anal Impact Questionnaire-7, *POPIQ-7* Pelvic Organ Prolapse Impact Questionnaire-7

**Table 5:** Pairwise comparison of changes over time within the cohort and between the cohorts; patient–reported pelvic floor outcomes (Pelvic Floor Distress Inventory 20 [PFDI–20], Pelvic Floor Impact Questionnaire – short form [PFIQ–7], and Pelvic Organ Prolapse/Urinary Incontinence Sexual Questionnaire [PISQ–12])

	Preoperatively to 3 months					3 months to 1 year					Preoperative to 1 year					^a^Differences in changes RASC vs Uphold™
	RASC		Uphold™			RASC		Uphold™			RASC		Uphold™			
	Mean ± SD	*n*	Mean ± SD	*n*	*p* value (RASC, Uphold™)	Mean ± SD	*n*	Mean ± SD	*n*	*p* value (RASC, Uphold™)	Mean ± SD	*n*	Mean ± SD	*n*	*p* value (RASC, Uphold™)	*p* value
PFDI-20 total	−49.5 ± 42.1	54	−67.5 ± 51.2	65	< 0.001	8 ± 33.6	53	−4.7 ± 33	59	0.393	−49.2 ± 43.7	55	−73 ± 55.6	63	< 0.001	0.005
					< 0.001					0.836					< 0.001	
POPDI–6	−27.7 ± 22.3	54	−38.7 ± 20.9	65	< 0.001	3.4 ± 14.5	53	−1 ± 13.5	59	0.270	−27 ± 23.9	56	−39.6 ± 23.6	63	< 0.001	< 0.001
					< 0.001					< 0.001					< 0.001	
CRADI–8	−6.7 ± 12.6	54	−10 ± 16.42	65	< 0.001	3.2 ± 14	53	0.3 ± 10.3	59	0.529	−6.6 ± 14.9	56	−11.2 ± 15.2	63	< 0.001	0.095
					< 0.001					0.999					< 0.001	
UDI–6	−15.1 ± 21.6	54	−18.8 ± 25.9	65	< 0.001	1.4 ± 17.9	53	−4 ± 20.8	59	0.3651	−15.5 ± 18.7	56	−22.2 ± 26.4	63	< 0.001	0.176
					< 0.001										< 0.001	
PFIQ–7 total	−38.4 ± 46.7	49	−42 ± 51.8	66	< 0.001	6.1 ± 29.2	51	3.1 ± 42.8	60	0.3231	−26.6 ± 45.1	52	−40.6 ± 52.8	63	< 0.001	0.296
					< 0.001										< 0.001	
PFIQ-UIQ-7	−9 ± 20.5	49	−14 ± 23.1	66	0.026	1.6 ± 16.1	51	−1.7 ± 15.9	60	0.991	−8.4 ± 18.4	52	−16.9 ± 24.4	63	0.008	0.093
					< 0.001										< 0.001	
PFIQ-CRAIQ-7	−3.3 ± 6.9	49	−2.4 ± 8.3	66	0.003	−1.9 ± 12.7	51	−3.7 ± 13.5	63	0.656	−5.9 ± 15.7	51	−6.6 ± 15.7	63	0.002	0.799
					0.008					0.069					0.004	
PFIQ-POPIQ-7	−16.1 ± 18.7	49	−13.6 ± 21.3	66	< 0.001	1.0 ± 12.3	50	−2.4 ± 17.5	59	10.682	−13.0 ± 21.2	51	−16.8 ± 24.4	62	< 0.001	0.541
					< 0.001										< 0.001	
PISQ–12 total	2.8 ± 7.2	36	−5.1 ± 13.7	36	0.431	−1.6 ± 6.7	28	8.6 ± 13.5	31	0.763	0.6 ± 9.4	33	1.7 ± 12.4	37	1	0.003
					0.094					0.007					0.680	
PISQ-12–behavioral	1.3 ± 2.6	35	−1.1 ± 3.7	35	0.042	−1.6 ± 2	28	1.4 ± 3.6	30	0.020	−0.2 ± 3	33	0.1 ± 2.9	37	1	< 0.001
					0.140					0.109					1	
PISQ-physical	2.3 ± 3.4	32	−0.2 ± 6.9	27	0.0121	0.6 ± 2.5	23	2.5 ± 5.3	23	0.349	2.9 ± 3.6	27	1.2 ± 6.8	32	0.004	0.348
										0.172					0.394	
PISQ–partner	0.4 ± 1.6	31	−0.2 ± 2.7	27	0.139	−0.6 ± 2.3	22	0.7 ± 2.2	23	0.502	−0.1 ± 1.9	27	−0.3 ± 2.4	29	1	0.023
					0.134					0.364					1	

Only patients reported the PFDI-20, PFIQ-7, and PISQ-12 at preoperatively, 3 months, and 1 year were included

Bonferroni post hoc test was used to compare changes within each surgery cohort over time: preoperatively, 3 months, and 1 year

ANOVA repeated measures was used to statistically test significant differences in changes over time between the two surgery cohorts

*POPDI-6* Pelvic Organ Prolapse/Urinary Incontinence Sexual Questionnaire-6, *CRADI-8* Colorectal-Anal Distress Inventory-8, *UDI-6* Urinary Distress Inventory-6, *UIQ-7* Urinary Impact Questionnaire-7, *CRAIQ-7* Colorectal-Anal Impact Questionnaire-7, *POPIQ-7* Pelvic Organ Prolapse Impact Questionnaire-7

^a^Between changes in the cohort = between changes, i.e., difference in the cohort scores from the preoperative period to the follow-up

Table 5 shows a sub-analysis of changes over time using a pairwise comparison in order to statistically investigate the reduction in the PFDI-20, PFIQ-7, and subscales scores comparing the cohorts over time. Using ANOVA repeated measures to compare changes over time, the only significant difference was the total PFDI-20 and the POPDI-6 subscale in the Uphold™ group compared with the RASC cohort (*p* = 0.005 and* p* < 0.001).

There was a relatively high loss to follow-up in reported PISQ-12 data. The available data indicated significant improvements in the total PISQ-12 for the Uphold™ cohort over time (*p* = 0.004), but no significance difference in the PISQ-12 subscales. In the RASC cohort, there was no significant improvement in the total PISQ-12 (*p* = 0.33), but there was a significant improvement in the PISQ-12 physical subscale over time (*p* = 0.005).

### Pain Outcome

Reported pain estimated by the VAS scale (0–10) was significantly improved from the preoperative to the 1-year assessment. In the RASC cohort, preoperative pain was estimated to 2.8 ± 2.8 and improved to 0.8 ± 1.7 at the 1-year follow-up (*p* < 0.001). Also, for the Uphold™ cohort, preoperative pain (2.2 ± 2.3) was significantly improved at the 1-year follow-up (0.5 ± 0.9), *p* < 0.001. No significant difference in pain was noted between the cohorts preoperatively (*p* = 0.16) or at the 1-year follow-up (*p* = 0.16).

### Sub-analysis of Covariates

We performed a multiple regression analysis to investigate if demographic factors influenced the 1-year improvement of the POP-Q anatomical outcomes and the reduction of total PFDI-20 and POPDI-6 scores. Only menopause had a significant confounding effect on the reduction of total PFDI-20 (*p* = 0.02) and the POPDI-6 (*p* = 0.02). To investigate if additional surgeries at the time of RASC or Uphold™ affected the 1-year POP-Q anatomical outcomes we carried out a logistic regression analysis and found that concurrent posterior colporrhaphy and perineorrhaphy had no significant influence on the apical POP-Q outcomes (*p* = 0.136, *p* = 0.999) or the total PFDI-20 and the POPDI-6 scores (*p* = 0.123, *p* = 0.962).

## Discussion

In this 1-year prospective study the main difference between RASC and Uphold™ for apical pelvic organ prolapse was that prolapse recurrence and reoperations for prolapse recurrence were more common after RASC. However, there were few and largely insignificant objective and subjective differences when comparing RASC and Uphold™. Mesh exposure was limited to 3 patients in the RASC group and 2 patients in the Uphold™ cohort, with a very low rate of serious complications overall.

Sacral colpopexy has previously been reported to have superior outcomes compared with a variety of vaginal procedures, including sacrospinous colpopexy, uterosacral colpopexy, and transvaginal mesh [5]. A growing body of evidence indicates efficacy of the Uphold™ mesh in restoring the pelvic floor anatomy and a recent randomized multicenter controlled study (ASPIRe) indicated that the composite primary efficacy of Uphold™ is non-inferior to the sacrocolpopexy (RASC, LSC, and abdominal procedures) [26, 27]. In the present study, anatomical success for the apical segment was achieved in 96.4% and 93.3% for the RASC and Uphold™ respectively, which confirms results from the ASPIRe study [26]. These results are also similar to previous reports suggesting that both methods might be highly efficacious in restoring pelvic floor anatomy in the short term and add to the growing body of literature in support of the Uphold™ vaginal mesh system [15, 16, 28].

In the ASPIRe study, additional concomitant prolapse procedures performed at the same time at which the sacrocolpopexy mesh was inserted were anterior repair (7%), posterior repairs/perineorrhaphy (47%), and MUS (43%) for stress urinary incontinence [26]. This is to be compared with only 7 perineorrhaphies and no MUS in the RASC cohort in the present study. The additional concomitant prolapse procedures may have led to a longer operation time in the ASPIRe study than in the RASC group in the present study (211.5 vs 150.8 min) [26]. There was also a shorter operation time for the Uphold™ procedure in the present study than in the ASPIRe study (68 vs 122 min) [26]. This may be partly explained by additional surgery by MUS in 42% and posterior repair in 63% of patients in the ASPIRe study compared with no MUS and 49% posterior repair in the present study [26]. However, the operation time for the Uphold™ in the present study was comparable with previously published studies [15, 16, 28].

In the present study, reoperation for any symptomatic recurrent prolapse 1 year after the RASC was 16%, which is comparable with 14% for the LSHP but higher if compared with 3% for sacrocolpopexy in the ASPIRe study [26, 28]. This may be because no additional concomitant anterior and posterior repairs were performed at the RASC cohort in the present study. The sacrocolpopexy anterior and posterior mesh strip extension is not standardized and may vary or has not been fully described in studies [6–8, 17–20, 26]. In the RASC cohort, the anterior mesh strip was adapted to the size of the prolapse, i.e., the defect in the anterior vaginal wall but up to a maximum extension of 4 cm long proximal to the vagina and cervix or proximal to the anterior vaginal wall if vault sacrocolpopexy. In other studies, a fixed anterior mesh strip length of 4 cm was described, whereas the posterior mesh strip may be extended down to the perineum [6–8, 28]. The downward extension of the anterior mesh strip may afford the anterior vaginal wall stability, resulting in a reduction of prolapse recurrence. However, this has been reported to carry a higher rate of urinary bladder and urethra injuries [6–8, 28]. The reoperation rate for symptomatic recurrent prolapse was comparatively low in the Uphold™ cohort (2.8%) and was similar to those of previous reports and also the 1-year result in the ASPIRe study (2%) [15, 16, 26]. The rate of concomitant vaginal surgery, such as anterior and posterior colporrhaphy, was low in the RASC cohort compared with the Uphold™ cohort. However, the concomitant vaginal surgery at the same time as the primary surgery had no statistically significant effect on the 1-year anatomical outcomes in the two cohorts. The optimal threshold for the learning curve for the RASC procedure is not clearly defined in the literature. These factors together may explain the differences in anatomical outcomes and reoperation rate for the RASC in the present and other studies. It is likely that standardization and/or combining vaginal prolapse surgery (supporting the prolapse levels II and III) with the RASC procedure may improve the anatomical outcomes and lower the reoperation rate for prolapse recurrence.

The RASC and the Uphold™ procedures inherently use different routes for apical prolapse surgery, the laparoscopic and the vaginal, yet both methods rely on polypropylene implants to create support. Although the robotic/laparoscopic/abdominal approaches provide full access to the apical segment and the posterior vaginal wall, access to repairing the middle and lower parts of the anterior wall is more limited. This may explain the higher reoperation rate in the anterior vaginal wall after RASC in the present study [28]. In contrast, using a vaginal approach allows for the repair of vaginal compartments and at all three prolapse levels. Surgery with the Uphold™ vaginal mesh may support the anterior vaginal wall down to the urethra level, i.e., pelvic organ level III support, which plausibly explains the low recurrence and reoperation rates of cystocele since spontaneous anterior repair.

Subjective pelvic floor outcomes (PFDI-20 and PFIQ-7, total and subscales) were also significantly improved for the two cohorts, with largely insignificant differences somewhat favoring the Uphold™ procedure. The Uphold™ cohort had a deterioration in the PISQ-12 partner domain but there was no overall deterioration or significant difference in sexual function between the cohorts. Preoperative pain, as estimated by a VAS scale, was significantly improved at follow-up visits for both RASC and Uphold™, with no significant differences between the cohorts. For Uphold™, improvement of pain has previously been reported by two studies using the same VAS pain scale at short- and long-term follow-up [10, 11, 15, 16]. This is in contrast to a previous report indicating no change in pain after LSHP [28]. These data suggest that use of vaginal mesh for prolapse repair may be dependent on the polypropylene load, surgical technique, and surgeon experience. Thus, mesh-based procedures for pelvic organ prolapse should be considered a heterogenous group of techniques that need to be evaluated individually when trying to determine lasting effects.

The overall intra- and postoperative complication rates were very low, also in comparison with previous studies, and showed no significant differences between RASC and Uphold™ [7, 8, 10–13, 15, 16, 24–29]. Only one intraoperative complication was reported in the RASC cohort (ureter injury) and one patient underwent mesh removal a few days after Uphold™. A large number of studies show that the risk for adverse events and complications is negatively associated with the volume of a specific procedure performed by the surgeon [27–32]. This association may provide support for the centralization of relatively infrequent procedures such as apical pelvic organ prolapse surgery [27, 30–33]. Taken together with the satisfactory objective and subjective outcomes for both procedures in the present study, our results suggest that choice of surgical method might be less important for the outcomes of apical prolapse surgery than surgical experience and surgical volume. It is beyond the scope of the present paper to address issues such as the definition of a high-volume center, allocation of resources needed to establish surgical centers and the health economic effects of centralization. However, in the case of RASC and Uphold™, centralization is more likely to yield satisfactory outcomes than inherent differences when choosing between the methods themselves.

In our study, women operated on using Uphold™ were more commonly postmenopausal and were on average 10 years older than those in the RASC cohort. Increasing age, and by association, menopausal status and overall morbidity, is likely to have affected the choice of performing surgery in spinal anesthesia and as a consequence choosing Uphold™ instead of RASC. Patients with preoperative recurrent prolapse were also significantly overrepresented in the Uphold™ cohort, whereas preoperative posterior vaginal wall prolapse was significantly overrepresented in the RASC cohort. Thus, Uphold™ may have been a logical choice for high-risk postmenopausal women and in the case of recurrent prolapse, whereas RASC was chosen for younger patients with more pronounced preoperative posterior vaginal wall prolapse. These choices are in line with previous findings that vaginal mesh may represent a good option for elderly high-risk women, whereas laparoscopic sacrocolpopexy is suitable for younger patients [24–26, 29, 33].

The present study was performed in a prospective controlled setting that was limited to three high-volume surgical centers with experienced surgeons. This is a strength of our study but at the same time a limitation because generalizability is limited. However, we prioritized internal validity in order for the results to be meaningful and relevant to a wider population under similar circumstances, i.e., high-volume centers. Other strengths included subjective and objective outcome assessments using validated instruments, cross-center standardized surgical procedures, and a relatively low loss to follow-up. We recognize that the lack of randomized surgical allocation is a major limitation of the study, which introduces unquantified bias. Despite our efforts to statistically adjust for differences in cohort demographic characteristics such as age and menopausal status (attributed to surgeon selection) a certain degree of bias cannot be ruled out. The short-term follow-up and the loss to follow-up may also limit the estimation of failure and re-intervention.

In summary, RASC and Uphold™ for apical pelvic organ prolapse are inherently different mesh-based procedures, which at high-volume centers are associated with satisfactory outcomes and low complication rates in the short term. Furthermore, there were few and largely insignificant objective and subjective differences when comparing the procedures. A long-term follow-up is required to determine if the favorable outcomes of the reoperation rate and the anterior vaginal wall anatomy shown in this study persist over time.

## References

[1] Barber MD, Maher C. Apical prolapse. Int Urogynecol J. 2013;24(11):1815–33. 24142057 10.1007/s00192-013-2172-1

[2] Benson JT, Lucente V, McClellan E. Vaginal versus abdominal reconstructive surgery for the treatment of pelvic support defects: a prospective randomized study with long-term outcome evaluation. Am J Obstet Gynecol. 1996;175(6):1418Y1421; discussion 1421Y1422. 8987919 10.1016/s0002-9378(96)70084-4

[3] Nygaard IE, McCreery R, Brubaker L, Connolly AM, Cundiff G, Weber AM, et al. Abdominal sacrocolpopexy: a comprehensive review. Obstet Gynecol. 2004;104(4):805Y823. 15458906 10.1097/01.AOG.0000139514.90897.07

[4] Geynisman-Tan J, Kenton K. Surgical updates in the treatment of pelvic organ prolapse. Rambam Maimonides Med J. 2017;8(2):e0017. 10.5041/RMMJ.10294 PMC5415363 28467763

[5] Maher C, Yeung E, Haya N, Christmann-Schmid C, Mowat A, Chen Z, Baessler K. Surgery for women with apical vaginal prolapse. Cochrane Database Syst Rev. 2023;7(7):CD012376. 37493538 10.1002/14651858.CD012376.pub2 PMC10370901

[6] Chang CL, Chen CH, Chang SJ. Comparing the outcomes and effectiveness of robotic-assisted sacrocolpopexy and laparoscopic sacrocolpopexy in the treatment of pelvic organ prolapse. Int Urogynecol J. 2022;33(2):297–308. 33760992 10.1007/s00192-021-04741-x

[7] Costantini E, Brubaker L, Cervigni M, Matthews CA, O’Reilly BA, Rizk D, Giannitsas K, Maher CF. Sacrocolpopexy for pelvic organ prolapse: evidence-based review and recommendations. Eur J Obstet Gynecol Reprod Biol. 2016;205:60–5. 27566224 10.1016/j.ejogrb.2016.07.503

[8] Culligan PJ, Saiz CM, Rosenblatt PL. Contemporary use and techniques of laparoscopic sacrocolpopexy with or without robotic assistance for pelvic organ prolapse. Obstet Gynecol. 2022;139(5):922–32. 35576354 10.1097/AOG.0000000000004761 PMC9015033

[9] FDA. 2019. https://www.fda.gov/news-events/press-announcements/fda-takes-action-protect-womens-health-orders-manufacturers-surgical-mesh-intended-transvaginal. Accessed 16 Apr 2019.

[10] Rahkola-Soisalo P, Mikkola TS, Altman D, Falconer C; Nordic TVM Group. Pelvic organ prolapse repair using the Uphold™ vaginal support system: 5-year follow-up. Female Pelvic Med Reconstr Surg. 2019;25(3):200–5. 29232268 10.1097/SPV.0000000000000530

[11] Falconer C, Altman D, Poutakidis G, Rahkola-Soisalo P, Mikkola T, Morcos E. Long-term outcomes of pelvic organ prolapse repair using a mesh-capturing device when comparing single- versus multicenter use. Arch Gynecol Obstet. 2020;303(1):135–42. 10.1007/s00404-020-05764-3. 32915305 10.1007/s00404-020-05764-3 PMC7854402

[12] Gillor M, Langer S, Dietz HP. A long-term comparative study of Uphold™ transvaginal mesh kit against anterior colporrhaphy. Int Urogynecol J. 2020;31(4):793–7. 31529327 10.1007/s00192-019-04106-5

[13] Allegre L, Debodinance P, Demattei C, Peray FP, Cayrac M, Fritel X, Courtieu C, Fatton B, de Tayrac R. Clinical evaluation of the Uphold™ LITE mesh for the surgical treatment of anterior and apical prolapse: a prospective, multicentre trial. Neurourol Urodyn. 2019;38(8):2242–9. 31359498 10.1002/nau.24125

[14] Lo TS, Pue LB, Tan YL, Hsieh WC, Kao CC, Uy-Patrimonio MC. Anterior-apical single-incision mesh surgery (Uphold™): 1-year outcomes on lower urinary tract symptoms, anatomy and ultrasound. Int Urogynecol J. 2019;30:1163–72. 30008078 10.1007/s00192-018-3691-6

[15] Altman D, Mikkola TS, Bek KM, Rahkola-Soisalo P, Gunnarsson J, Engh ME, Falconer C; Nordic TVM group. Pelvic organ prolapse repair using the Uphold™™ Vaginal Support System: a 1-year multicenter study. Int Urogynecol J. 2016;27(9):1337–45. 26874525 10.1007/s00192-016-2973-0

[16] Morcos E, Altman D, Hunde D, Falconer C, Nordic TVM group. Comparison of single- versus multicenter outcomes for pelvic organ prolapse repair using a mesh-capturing device. Int Urogynecol J. 2018;29(1):91–7. 28547270 10.1007/s00192-017-3364-x

[17] Ramm O, Kenton K. Robotic/laparoscopic prolapse repair: role of hysteropexy: a urogynecology perspective. Urol Clin North Am. 2012;39(3):343–8. 22877717 10.1016/j.ucl.2012.06.008

[18] Pan K, Cao L, Ryan NA, Wang Y, Xu H. Laparoscopic sacral hysteropexy versus laparoscopic sacrocolpopexy with hysterectomy for pelvic organ prolapse. Int Urogynecol J. 2016;27(1):93–101. 26179552 10.1007/s00192-015-2775-9

[19] Ganatra AM, Rozet F, Sanchez-Salas R, Barret E, Galiano M, Cathelineau X, Vallancien G. The current status of laparoscopic sacrocolpopexy: a review. Eur Urol. 2009;55(5):1089–103. 19201521 10.1016/j.eururo.2009.01.048

[20] Price N, Slack A, Jackson SR. Laparoscopic hysteropexy: the initial results of a uterine suspension procedure for uterovaginal prolapse. BJOG. 2010;117(1):62–8. 20002370 10.1111/j.1471-0528.2009.02396.x

[21] Vu MK, Letko J, Jirschele K, Gafni-Kane A, Nguyen A, Du H, et al. Minimal mesh repair for apical and anterior prolapse: initial anatomical and subjective outcomes. Int Urogynecol J. 2012;23:1753–61. 22531956 10.1007/s00192-012-1780-5

[22] Bump RC, Mattiasson A, Bø K, Brubaker LP, DeLancey JO, Klarskov P, Shull BL, Smith AR. The standardization of terminology of female pelvic organ prolapse and pelvic floor dysfunction. Am J Obstet Gynecol. 1996;175(1):10–7. 8694033 10.1016/s0002-9378(96)70243-0

[23] Barber MD, Walters MD, Bump RC. Short forms of two condition-specific quality-of-life questionnaires for women with pelvic floor disorders (PFDI-20 and PFIQ-7). Am J Obstet Gynecol. 2005;193(1):103–13. 16021067 10.1016/j.ajog.2004.12.025

[24] Rogers RG, Kammerer-Doak D, Villarreal A, Coates K, Qualls C. A new instrument to measure sexual function in women with urinary incontinence or pelvic organ prolapse. Am J Obstet Gynecol. 2001;184(4):552–8. 11262452 10.1067/mob.2001.111100

[25] Wagemakers SH, van der Velden JM, Gerlich AS, Hindriks-Keegstra AW, van Dijk JFM, Verhoeff JJC. A systematic review of devices and techniques that objectively measure patients’ pain. Pain Physician. 2019;22(1):1–13. 30700064

[26] Menefee SA , Richter HE, Myers D, Moalli P, Weidner AC, Harvie HS, Rahn DD, Meriwether KV, Paraiso MFR, Whitworth R, Mazloomdoost D, Thomas S; NICHD Pelvic Floor Disorders Network. Apical suspension repair for vaginal vault prolapse: a randomized clinical trial. JAMA Surg. 2024;159(8):845–55. 10.1001/jamasurg.2024.1206. 38776067 10.1001/jamasurg.2024.1206 PMC11112501

[27] Geoffrion R, Larouche M. Guideline No. 413: Surgical Management of Apical Pelvic Organ Prolapse in Women. J Obstet Gynaecol Can. 2021;43(4):511–23.e1. 33548503 10.1016/j.jogc.2021.02.001

[28] Gutman RE, Rardin CR, Sokol ER, Matthews C, Park AJ, Iglesia CB, Geoffrion R, Sokol AI, Karram M, Cundiff GW, Blomquist JL, Barber MD. Vaginal and laparoscopic mesh hysteropexy for uterovaginal prolapse: a parallel cohort study. Am J Obstet Gynecol. 2017;216(1):38.e1–11. 27596620 10.1016/j.ajog.2016.08.035

[29] Ng-Stollmann N, Fünfgeld C, Gabriel B, Niesel A. The international discussion and the new regulations concerning transvaginal mesh implants in pelvic organ prolapse surgery. Int Urogynecol J. 2020;31(10):1997–2002. 32696186 10.1007/s00192-020-04407-0 PMC7497328

[30] Lundström NR, Berggren H, Björkhem G, Jögi P, Sunnegârdh J. Centralization of pediatric heart surgery in Sweden. Pediatr Cardiol. 2000;21(4):353–7. 10865012 10.1007/s002460010079

[31] Chan JK, Gardner AB, Taylor K, Blansit K, Thompson CA, Brooks R, Yu X, Kapp DS. The centralization of robotic surgery in high volume centers for endometrial cancer patients—a study of 6560 cases in the U.S. Gynecol Oncol. 2015;138(1):128–32. 25933680 10.1016/j.ygyno.2015.04.031

[32] Morcos E, Falconer C, Grip ET, Geale K, Hellgren K, Poutakidis G, Altman D. Association between surgical volumes and real-world healthcare cost when using a mesh capturing device for pelvic organ prolapse: a 5-years comparison between single- versus multicenter use. Int Urogynecol J. 2021;32(11):3007–15. 33635348 10.1007/s00192-021-04698-x PMC8536564

[33] Obinata D, Sugihara T, Yasunaga H, Mochida J, Yamaguchi K, Murata Y, Yoshizawa T, Matsui T, Hiroki Matsui H, Sasabuchi Y, Fujimura T, Homma Y, Takahashi S. Tension-free vaginal mesh surgery versus laparoscopic sacrocolpopexy for pelvic organ prolapse: analysis of perioperative outcomes using a Japanese national inpatient database. Int J Urol. 2018;25(7):655–9. 29729035 10.1111/iju.13587

